# Brain and renal oxygenation measured by NIRS related to patent ductus arteriosus in preterm infants: a prospective observational study

**DOI:** 10.1186/s12887-021-03036-w

**Published:** 2021-12-09

**Authors:** Jurate Navikiene, Ernestas Virsilas, Ramune Vankeviciene, Arunas Liubsys, Augustina Jankauskiene

**Affiliations:** Faculty of Medicine, Institute of Clinical Medicine, Vilnius University, Vilnius, Lithuania

**Keywords:** Patent ductus arteriosus, Preterm infant, Cerebral oxygenation, Renal oxygenation, Near-infrared spectroscopy

## Abstract

**Background:**

Patent ductus arteriosus (PDA) is common among preterm neonates. Haemodynamically significant ductus arteriosus (hsPDA) can cause ductal steal and contribute to poor outcomes. Our aim was to evaluate ductus arteriosus patency and significance using two-site near-infrared spectroscopy (NIRS) measurements in preterm infants older than 72 h as a supplemental tool to echocardiography.

**Methods:**

In this prospective observational study, 123 preterm infants (gestational age (GA) < 32 weeks, birth weight < 1500 g) were enrolled. Sixty-four newborns had closed ductus arteriosus (noPDA), and 41 and 18 patients were assigned to the PDA and hsPDA groups, respectively, per predefined echocardiographic criteria. Cerebral and renal oxygenation were assessed during NIRS monitoring.

**Results:**

A higher renal mean (±SD) regional tissue oxygen saturation (rSpO_2_) (76.7 (±7.64)) was detected in the noPDA group than in the PDA (71.7 (±9.02)) and hsPDA (67.4 (±13.48)) groups (*p* < 0.001). Renal fractional tissue oxygen extraction (FTOE) (0.18 (±0.079)) was lower in the noPDA group than in the PDA (0.23 (±0.092)) and hsPDA (0.24 (±0.117))0.117 groups (*p* = 0.002). Cerebral oxygenation was significantly lower in the hsPDA group (77.0 (±5.16)) than in the noPDA (79.3 (±2.45)) and PDA (79.7 (±2.27)) groups (*p* = 0.004). There was no significant difference in cerebral fractional tissue oxygen extraction (FTOE) between any of the groups.

**Conclusions:**

Our results suggest that renal oxygenation is affected by ductus patency in preterm infants older than 72 h. Significant differences in cerebral oxygenation were observed between the hsPDA group and the PDA and noPDA groups.

**Trial registration:**

ClinicalTrials.gov Identifier: NCT04295395. Registration date: 4 March 2020. This study was retrospectively registered, https://clinicaltrials.gov/ct2/show/NCT04295395.

## Background

The prevalence rates of patent ductus arteriosus (PDA) among preterm infants at four days of age are approximately 10% among infants born at 30 through 37 weeks of gestation, 80% among those born at 25 through 28 weeks of gestation, and 90% among those born at 24 weeks of gestation [1].

The presence of PDA can result in the ductal steal phenomenon, also known as haemodynamically significant PDA (hsPDA), leading to pulmonary hyperperfusion and systemic hypoperfusion. The ductal steal phenomenon reduces cerebral and renal regional blood flow and increases the risk of pulmonary and intraventricular haemorrhage (IVH), necrotizing enterocolitis (NEC), acute kidney injury, bronchopulmonary dysplasia (BPD), and death [2–6].

Echocardiography, a noninvasive and relatively simple method for the diagnosis of ductus arteriosus (DA) patency at the bedside, is widely used in the neonatal population. It can address anatomical DA features (ductal size and left atrium/aortic root ratio (LA/Ao)) and haemodynamic effects (shunt direction and size) by blood flow Doppler. However, echocardiography requires experience and specific training, and often, it cannot be performed in a timely manner by paediatric cardiologists.

The haemodynamic significance of PDA, a very important factor, should be interpreted by considering gestational and chronological age and other possible contributors; however, debates about the most appropriate method of identification and even definition of hsPDA are still ongoing [7]. More importantly, it is currently unknown how hsPDA influences regional blood flow in the most vulnerable organs of premature infants, namely, the brain, kidneys, and intestine, and existing clinical research is controversial.

Near-infrared spectroscopy (NIRS) is a noninvasive clinical tool that can provide continuous and real-time information regarding regional tissue oxygen saturation (rSpO_2_) and indirectly information on regional organ perfusion. It is based on the relative transparency of biological tissues (bone, skin, and soft tissue) and the ability to differentiate oxygenated haemoglobin from deoxygenated haemoglobin, as they have distinct absorption spectra. The ratio of oxygenated and total haemoglobin represents the rSpO_2_ [8]. Numerous clinical studies have been conducted to determine normal NIRS values for preterm neonates and to assess the use of NIRS to evaluate the regional brain and kidney oxygenation in different pathologic conditions, including PDA [9, 10]. Despite the growing clinical use of cerebral and peripheral NIRS monitoring in premature infants, its routine use and importance in determining PDA significance is still controversial [11–13].

The aim of this study was to evaluate DA patency and significance using two-site NIRS measurements in preterm infants older than 72 h as a supplemental tool to echocardiography. We hypothesized that hsPDA results in significant cerebral and renal hypoperfusion, which is reflected by NIRS measurements.

## Methods

### Patients

A prospective observational centre-based study was carried out in a tertiary-level NICU. The study was approved by the regional ethics committee and registered at clinicaltrials.com (reg. No. NCT04295395). Informed parental consent was obtained before enrolment. Very low birth weight (VLBW) (< 1500 g) infants with gestational ages (GAs) less than 32 weeks and older than 72 h who underwent echocardiographic screening for PDA were included in the study. The exclusion criteria were major congenital anomalies, the need for cardiovascular support with vasopressor medication and culture-proven sepsis.

### Study design

Study participants were classified into 3 groups: infants with no PDA (1), infants with haemodynamically insignificant PDA (2) and infants with hsPDA (3). According to the clinical protocol used in our NICU, haemodynamic significance was determined by echocardiographic ductal left-to-right shunt, volume overload according to a LA/Ao ratio ≥ 1.4 and DA size > 2 mm. Echocardiography was performed after the 3rd day of life to avoid possible bidirectional shunting during the transitional period. Patients with open DA without the aforementioned haemodynamic significance criteria were assigned to the PDA group. All echocardiographic studies were performed by a paediatric cardiologist. DA was evaluated from a high parasternal view using colour Doppler assessment. The DA diameter was measured at the narrowest point. The LA/Ao ratio was measured in the parasternal long axis view using the M-Mode. Ductal closure was documented by no ductal blood flow on colour Doppler scanning. If echocardiography showed closed DA or ductus patency, the measurements were considered valid for 24 h. The resistive index (RI) was evaluated in the anterior cerebral artery (ACA) using pulsed-wave Doppler on the day of NIRS monitoring. *Data collection*: Perinatal and demographic data were collected, and cranial baseline ultrasound data were obtained after enrolment. NIRS (NONIN SenSmart, X-100, USA) was used for two-site regional tissue oxygenation measurements. Neonatal/paediatric sensors (SenSmart 8004CB-NA non-adhesive with EQUANOXTM technology) were applied to the forehead lateral to the midline, superior to the eyebrow and inferior to the hairline on either the left or right posterior flank above the iliac crest and below the costal margin to measure cerebral and renal rSpO_2_, respectively, using a wrap to secure the sensor. The NIRS apparatus was left in place for 12 h for continuous data acquisition. Recordings were briefly interrupted every 3 h to reposition the sensor medially or laterally to prevent skin bruising or breakdown. During data recording, minimal-handling nursing care guidelines were followed [14]. All 12 h of gathered data were analysed in every group except the hsPDA group, where 1 h of analysis was used (for prompt treatment initiation). Simultaneously, the SpO_2_ was measured to calculate the fractional tissue oxygen extraction (FTOE), which reflects the balance between tissue oxygen delivery and tissue oxygen consumption, indirectly reflecting tissue perfusion [15]. All groups had the same saturation target of 89–95% with the lowest possible FiO_2_ and with lower and upper alarm limits of 88 and 96%, respectively. Cerebral-to-renal oxygenation ratio (CROR) was calculated from regional renal and cerebral tissue oxygenation levels. For each studied infant, data were collected on GA, birth weight, sex, type of delivery, antenatal steroids, Apgar scores at 1 and 5 min, surfactant administration, blood pH, lactate, platelet count, haemoglobin, haematocrit, FiO_2_ requirement at enrolment, need for respiratory support (noninvasive (nasal continuous positive airway pressure (NCPAP)) or invasive (mechanical ventilation)), and urine output. *Statistical analysis*: The frequency and mean (standard deviation) are used to describe quantitative and qualitative data, respectively. The chi-squared test was used to compare quantitative parameters between subject groups. One-way ANOVA was used to evaluate the mean differences among subject groups. Post hoc analysis with Bonferroni adjustment for multiple comparisons was used for pairwise comparisons. A two-tailed *p*-value less than 0.05 was considered to be significant. Statistical analysis was performed using Statistical Analysis System (SAS) package version 9.2.

## Results

**Fig. 1:**
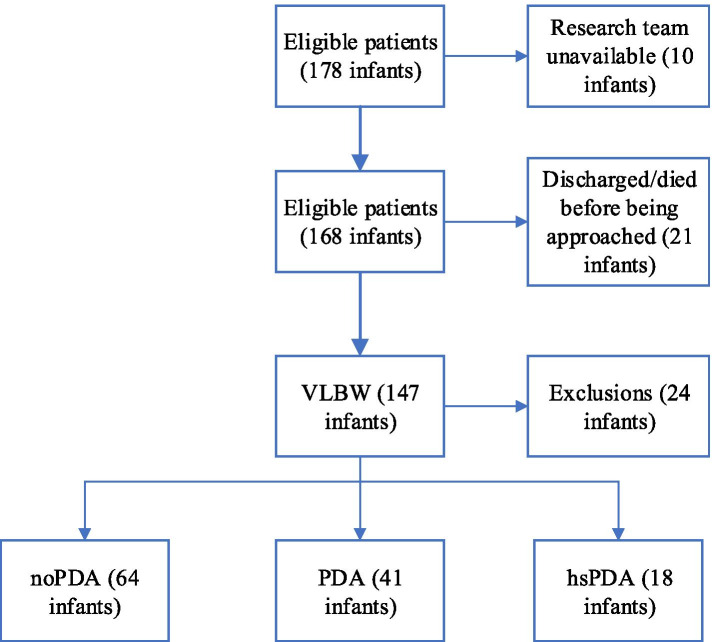
Consort diagram of the study

A total of 147 patients met the inclusion criteria, and 24 patients were excluded due to consent withdrawal (*n* = 10), congenital malformations (*n* = 5) and sepsis/haemodynamic instability (*n* = 9) (Fig. 1).

Sixty-four newborns had no PDA, and 41 and 18 patients were assigned to the PDA and hsPDA groups, respectively. General patient characteristics are shown in Table 1.

**Table 1:** General characteristics

	noPDA (*n* = 64)	PDA (*n* = 41)	HsPDA (*n* = 18)	*p* value
Gestational age (weeks), mean (SD)^1–3^	28.5 (1.92)	27.2 (1.65)	25.8 (2.01)	< 0.001
Birth weight (g), mean (SD)^1–3^	1118.6 (221.03)	1009.5 (215.43)	839.4 (219.99)	< 0.001
Male, %	31 (48.4)	23 (56.1)	10 (55.6)	NS
Apgar at 1 min, mean (SD)	7 (1.62)	6.7 (1.82)	5.8 (2.28)	NS
Apgar at 5 min, mean (SD)	8 (1.41)	7.9 (1.19)	7.4 (1.33)	NS
Hours of life at measurement, mean (SD)^2, 3^	150.4 (45.3)	141.6 (36.7)	185.8 (58.0)	0.003
Haematocrit (%), mean (SD)^2^	46.03 (8.48)	43.67 (10.523)	38.71 (7.094)	0.011
Platelets (× 10^9^/l), mean (SD)	269.19 (99.687)	254 (116.908)	258.44 (150.983)	NS
pCO_2_, mean (SD)	42.25 (6.816)	44.41 (9.25)	46.09 (10.912)	NS
Lactate (mmol/l), mean (SD)	1.94 (0.598)	2.15 (1.084)	2.06 (0.523)	NS
Urine output (ml/kg/h), mean (SD)	3.91 (0.796)	3.63 (0.971)	3.51 (0.899)	NS
Antenatal steroids 2x, %	42 (66.7)	32 (78.0)	13 (72.2)	NS
Chorioamnionitis, %	16 (25.8)	11 (28.9)	6 (35.3)	NS
Caesarean section, %	44 (68.8)	33 (80.5)	8 (44.4)	0.025
Surfactant, %	41 (64.1)	36 (87.8)	17 (94.4)	0.003
Mechanical ventilation, %	11 (17.2)	18 (43.9)	16 (88.9)	< 0.001
Non-invasive respiratory support, %	35 (54.7)	17 (41.5)	1 (5.6)	< 0.001
FiO_2_, mean (SD)^2, 3^	0.25 (0.05)	0.25 (0.05)	0.32 (0.09)	< 0.001
Ductus diameter (cm), mean (SD)^3^	–	0.17 (0.039)	0.26 (0.032)	< 0.001
LA/Ao ratio, mean (SD)^1–3^	1.24 (0.174)	1.38 (0.202)	1.72 (0.179)	< 0.001
RI ACA, mean (SD)^1–3^	0.7 (0.039)	0.74(0.061)	0.84 (0.144)	< 0.001
CROR, mean (SD)^1, 2^	0.97 (0.091)	0.90 (0.108)	0.87 (0.167)	0.001

1: Statistically significant difference between the “noPDA” and “PDA” groups

2: Statistically significant difference between the “noPDA” and “HsPDA” groups

3: Statistically significant difference between the “PDA” and “HsPDA” groups. NS: not significant

There was no significant difference in sex, Apgar score, platelet count or urine output among the groups. GA and birth weight were lowest, while surfactant administration rates and oxygen requirements were highest in the HsPDA group and differed significantly.

Patients without DA had higher renal rSpO_2_ and lower renal FTOE values than the PDA and hsPDA groups. The cerebral rSpO_2_ was significantly lower in the hsPDA group than in the other groups (Table 2).

**Table 2:** NIRS and SpO_2_ measurements

	noPDA (*n* = 64) Mean (SD)	PDA (n = 41) Mean (SD)	HsPDA (n = 18) Mean (SD)	P value
Cerebral rSpO_2_^2, 3^	79.3 (2.45)	79.7 (2.27)	77.0 (5.16)	0.004
Renal rSpO_2_^1, 2^	76.7 (7.64)	71.7 (9.02)	67.4 (13.48)	< 0.001
SpO_2_^2, 3^	93.8 (1.88)	93.7 (1.62)	91.7 (2.70)	0.001
Cerebral FTOE	0.15 (0.033)	0.15 (0.026)	0.16 (0.047)	0.566
Renal FTOE^1, 2^	0.18 (0.079)	0.23 (0.092)	0.24 (0.117)	0.002

1: Statistically significant difference between the “noPDA” and “PDA” groups

2: Statistically significant difference between the “noPDA” and “HsPDA” groups

3: Statistically significant difference between the “PDA” and “HsPDA” groups

There was no significant difference in cerebral FTOE among the groups. The ductal diameter and LA/Ao ratio were significantly correlated with renal FTOE and renal rSpO_2_ but not with cerebral NIRS measurements. Spearman’s correlation coefficients between echocardiographic parameters of haemodynamic significance and NIRS measurements are presented in Table 3.

**Table 3:** Correlation between echocardiography parameters and NIRS measurements

	Cerebral FTOE	Renal FTOE	Cerebral rSpO_2_	Renal rSpO_2_	SpO_2_	CROR
Ductal diameter	−0.02	0.36***	−0.08	−0.39***	− 0.17	−0.37***
LA/Ao ratio	−0.08	0.23*	−0.05	−0.27***	− 0.21*	−0.27*
RI ACA	0.03	0.24*	−0.18*	−0.24*	− 0.23*	−0.16

* *p* < 0.05

*** *p* < 0.01

## Discussion

Our study demonstrates that in VLBW preterm infants (< 32 weeks of gestation) older than 72 h of life cerebral oxygen saturation is significantly lower in infants with echocardiographic signs of highly significant PDA compared to patients with no PDA and closed DA. We also demonstrated that renal regional oxygenation is higher in patients with no PDA or closed DA than in infants with PDA and hsPDA. In contrast, cerebral oxygenation in infants with hemodynamically insignificant PDA and was not found to be different from that of neonates with closed DA. Regional oxygenation reflects the amount of oxygen used by the tissue. Our study found significantly lower cerebral rSpO_2_ in the hsPDA group. This finding is contrary to those of several studies, which found no difference in cerebral regional oxygenation in the groups [11, 13, 16, 17]. One explanation of this finding could be that hsPDA group patients had lower SpO_2_ despite receiving more supplemental oxygen and invasive respiratory support. However, in our study, cerebral FTOE was not significantly different in the groups, which is consistent with the aforementioned studies. In theory, lower cerebral rSpO_2_ should result in higher cerebral FTOE, which, in accordance with published research, reflects impaired cerebral perfusion [18, 19]. In contrast, intact cerebral autoregulation ensures stable blood flow despite blood pressure fluctuations. However, previous studies established that autoregulation capacity in preterm neonates is reduced to an unknown extent [20]. Additionally, large PDA in preterm infants negatively affects brain circulation, which could result in lower regional cerebral oxygenation [21]. In our study, the hsPDA group was composed of the smallest and “sickest” infants, the majority of whom required mechanical ventilation. Previous research indicates that respiratory distress syndrome (RDS) predisposes patients to a lack of cerebral autoregulation, which might explain the absence of a correlation between cerebral rSpO_2_ and FTOE in our patients [22]. However, Schwarz et al. [23] found higher cerebral FTOE without lower rSpO_2_, and Arman et al. [24] and Poon & Tagamolila [25] found significantly lower cerebral rSpO_2_ and higher cerebral FTOE in VLBW preterm infants with hsPDA before medical and/or surgical closure of PDA.

Our study also showed that neonates with PDA and hsPDA have significantly lower renal saturation levels and higher FTOE than infants with no PDA. These findings are contrary to those of Chock VY et al., where renal saturation below 66% was associated with hsPDA [11]. We speculate that PDA, regardless of DA size and haemodynamic significance, has a negative impact on renal blood flow. Although statistical significance was not achieved, van der Laan also reported higher renal saturation and lower renal FTOE in infants without PDA than in those with PDA and hsPDA, a result that is parallel to ours [13]. This finding might be further implied in a study performed by Petrova et al. that demonstrated no renal regional saturation or renal FTOE differences between moderate- and large-sized PDAs [16]. These findings are conclusive representing the ductal steel phenomenon leading to a significantly reduced blood flow distal of the PDA including the kidneys. In contrast, preserved cerebral autoregulation likely explains the greater sensitivity of renal rSpO_2_ and FTOE than cerebral measurements in infants with hsPDA [11]. Despite the effects on renal saturation and FTOE, urine output was within the normal range in all three groups. We did not find any correlation between the RI of the ACA and cerebral rSpO_2_, but a study by Arman et al. found that Doppler and NIRS measurements correlated significantly [24].

There are several possible reasons for these inconsistent findings. Some of the studies evaluated only ductal size and did not evaluate flow pattern and/or possible volume overload [16, 17]. Moreover, there were significant variations of NIRS application times among studies – most used NIRS up to 1 h, and only Chock et al., Arman et al. and Poon & Tagamolila used NIRS for 24 h or more. Additionally, in some studies, infants receiving vasopressor medications were included in hsPDA groups more often than in control groups, which could influence cerebral oxygenation [17, 26]. Furthermore, the timing of inclusion differed among studies, and most performed NIRS monitoring in the first 72 h after birth, which might be considered a transitional period [13, 16]. Finally, as is typical in neonatal research, most of the studies consisted of relatively small sample sizes, which could reduce the ability to obtain significant differences.

Other findings associated with hsPDA or PDA were not different from previous findings. The lactate level did not differ among the groups, as previous studies demonstrated it to be a poor indicator of PDA [27]. Platelet counts were not different among our groups, although some studies have indicated a marginal association between PDA and low platelet counts in the first days of life [28]. This result was not found, possibly because our patients were beyond the transitional period. As described in previous research, hsPDA patients had a higher oxygen requirement and an increased rate of mechanical ventilation [7, 29]. However, this could be attributed to other factors, such as a lower birth weight and GA, as some studies report no differences between the noPDA and PDA groups [30, 31].

We acknowledge the limitations of our study. First, we did not obtain fluid therapy data before inclusion, which could have influenced the grouping of the sample. We also chose to evaluate PDA after a transitional 3-day period to avoid the confounding bias caused by neonatologists, rather than paediatric cardiologists, assessing the haemodynamic significance and possible closure of PDA. Furthermore, the neonates in the studied groups differed significantly, and the hsPDA group consisted of infants with the lowest GAs and birth weights. However, this inverse correlation with GA and birth weight is well known and to be expected given the relatively large sample size [32, 33]. Second, patients were included within a single institution that favours aggressive management of hsPDA rather than awaiting possible spontaneous ductal closure. Finally, the hsPDA group size was relatively small and may have been underrepresented. Nonetheless, our study findings show that cerebral and renal regional NIRS measurements may supply additional information for h sPDA evaluation and treatment decisions. Further prospective multicentre studies could clarify the role of NIRS in PDA management. Our study also has some strengths as well. All infants received echocardiographic evaluations, which included examinations of ductal size, flow pattern and the LA/Ao ratio, while some of the previous studies included only ductal size as a sole determinant of haemodynamic significance. None of the recruited patients received vasoactive medications, which might impair cerebral and/or renal autoregulation. Finally, all data were collected prospectively.

## Conclusions

In our cohort, we found a statistically significant difference in cerebral oxygenation between the hsPDA group and the PDA and noPDA groups. Furthermore, we suggest that renal rSpO_2_ and FTOE are affected earlier by ductus presence and are more sensitive to ductal steal than cerebral rSpO_2_. The authors conclude that NIRS can be used as a supplementary tool to provide additional information in VLBW, < 32-week GA, preterm infants older than 72 h regarding treatment initiation.

## Data Availability

The datasets used and/or analysed during the current study are available from the corresponding author upon reasonable request.

## Abbreviations

ACA: anterior cerebral artery
BPD: bronchopulmonary dysplasia
CROR: cerebral-to-renal oxygenation ratio
DA: ductus arteriosus
FTOE: fractional tissue oxygen extraction
GA: gestational age
hsPDA: haemodynamically significant ductus arteriosus
IVH: intraventricular haemorrhage
LA/Ao: left atrium/aortic root ratio
NCPAP: nasal continuous positive airway pressure
NEC: necrotizing enterocolitis
NIRS: near-infrared spectroscopy
PDA: patent ductus arteriosus
RDS: respiratory distress syndrome
RI: resistive index
rSpO2: regional tissue oxygen saturation
VLBW: very low birth weight
